# Insight into Salivary Gland Aquaporins

**DOI:** 10.3390/cells9061547

**Published:** 2020-06-25

**Authors:** Claudia D’Agostino, Osama A. Elkashty, Clara Chivasso, Jason Perret, Simon D. Tran, Christine Delporte

**Affiliations:** 1 Laboratory of Pathophysiological and Nutritional Biochemistry, Faculty of Medicine, Université Libre de Bruxelles, 808 Route de Lennik, Blg G/E CP 611, B-1070 Brussels, Belgium; Claudia.DAgostino@ulb.be (C.D.); clara.chivasso@ulb.ac.be (C.C.); jason.perret@ulb.ac.be (J.P.); 2 McGill Craniofacial Tissue Engineering and Stem Cells Laboratory, Faculty of Dentistry, McGill University, Montreal, QC H3A 0C7, Canada; osama.elkashty@mail.mcgill.ca (O.A.E.); simon.tran@mcgill.ca (S.D.T.); 3 Oral Pathology Department, Faculty of Dentistry, Mansoura University, 35516 Mansoura, Egypt

**Keywords:** aquaporin, salivary gland, physiology, pathophysiology, regenerative medicine, xerostomia

## Abstract

The main role of salivary glands (SG) is the production and secretion of saliva, in which aquaporins (AQPs) play a key role by ensuring water flow. The AQPs are transmembrane channel proteins permeable to water to allow water transport across cell membranes according to osmotic gradient. This review gives an insight into SG AQPs. Indeed, it gives a summary of the expression and localization of AQPs in adult human, rat and mouse SG, as well as of their physiological role in SG function. Furthermore, the review provides a comprehensive view of the involvement of AQPs in pathological conditions affecting SG, including Sjögren’s syndrome, diabetes, agedness, head and neck cancer radiotherapy and SG cancer. These conditions are characterized by salivary hypofunction resulting in xerostomia. A specific focus is given on current and future therapeutic strategies aiming at AQPs to treat xerostomia. A deeper understanding of the AQPs involvement in molecular mechanisms of saliva secretion and diseases offered new avenues for therapeutic approaches, including drugs, gene therapy and tissue engineering. As such, AQP5 represents a potential therapeutic target in different strategies for the treatment of xerostomia.

## 1. Introduction

Salivary glands (SG) consist of three pairs of major SG, i.e., submandibular (SMG), parotid (PG) and sublingual (SLG) glands, and minor SG (MSG) scattered throughout the mouth cavity. SG are made of several cell types: acinar cells (secretory cells), ductal cells, myoepithelial cells surrounding the acinar cells and endothelial cells. SG play an important role in human health homeostasis by secreting about 0.5 to 1.5 L of saliva daily [1]. Saliva is a body fluid containing numerous compounds, playing a crucial role in food handling (bolus formation, taste, digestion), defense against microorganisms (e.g., virus, fungi and bacteria), and protection of teeth (buffering, lubrication, remineralization, protection against demineralization) [2]. Among the numerous saliva components, water is by far the component present in the highest proportion. The secretion of saliva, including water transport, is explained by a two-step secretory mechanism. In the first step, the SG acinar cells secrete an important concentration of sodium chloride into the lumen of acinar cell aggregates, called acini, leading to the formation of trans-epithelial sodium chloride gradient, subsequent trans-epithelial water transport from the acini to the acini lumen and the formation of a primary isotonic fluid. In the second step, this primary isotonic fluid flows through the lumen of the ducts where the ductal cells reabsorb part of the sodium chloride and secrete bicarbonate. Due to the water-impermeable characteristics of the ductal cells, water does not follow the reabsorption of NaCl. Thereby, the final saliva flowing into the mouth cavity is hypotonic [3].

Transcellular water permeability is ensured by a family of water channels called aquaporins (AQPs) [4,5]. AQPs are small transmembrane proteins of molecular weight ranging from approximately 27 to 37 kDa. AQPs possess six transmembrane helices and two short helices containing a signature motif Asparagine-Proline-Alanine involved in the establishment of the water pore, forming an ‘hourglass-like’ structure [5]. While it was thought for many years that the association into tetramers was required for proper AQPs function [6], monomeric AQPs may be able to retain function [7,8]. Still, the reason for AQP tetramerization remains an open question. In mammals, AQPs can be subdivided into classical AQPs permeable only to water (AQP1, AQP2, AQP4, AQP5, AQP6, AQP8), aquaglyceroporins permeable to small solutes like glycerol and urea in addition to water (AQP3, AQP7, AQP9, AQP10) and unorthodox AQPs with undefined permeability (AQP11, AQP12) [5,9,10]. Noteworthy, however, is that the unorthodox AQP11 is glycerol permeable [11]. Several AQPs are expressed by the various SG cells [12,13], including AQP5 that plays an important role in the mechanism of saliva secretion [14,15,16] 

This review will provide an insight into SG AQPs by addressing their cellular distribution, physiological function, involvement in SG pathologies, and the therapeutic strategies aiming at AQPs.

## 2. Cellular Distribution of AQPs in Adult SG Subsection

### 2.1. Human

In human SG, AQP1 expression is restricted to vascular endothelium and myoepithelial cells surrounding the acini [17,18,19]. AQP3 expression was detected on the basolateral membranes of both serous and mucous acinar cells and ductal cells [18,20]. Immunohistological studies revealed that AQP4 expression was located at the basal region of acinar cells, at the apicolateral membrane of intercalated and striated ducts, and in myoepithelial cells [21]. AQP5 protein is predominantly expressed at the apical membrane, with slight expression at the basolateral membrane of serous and mucous acini [12,19,22]. In contrast to intercalated ductal cells expressing AQP5 during SG development, mature ducts are devoid of AQP5 labeling [18]. To the best of our knowledge, protein expression of the other AQPs has not been documented in human SG.

### 2.2. Mouse

AQP1 is expressed in myoepithelial and endothelial cells of mouse SG, but not in acinar and ductal cells [20,23]. AQP3, AQP4 and AQP8 are located at the basal region of acinar cells and in ductal cells [13,21,23]. AQP8 is also expressed by myoepithelial cells [24]. Both the apical and basolateral membranes of acinar cells display positive AQP5 labeling [17,22,25,26]. In addition, intercalated ducts express AQP5 at their apical membrane [27]. AQP7 is localized in endothelial cells [23]. The superaquaporin AQP11 was found in the ductal epithelia of both young and adult mice [23,28].

### 2.3. Rat Subsection

**Figure 1:**
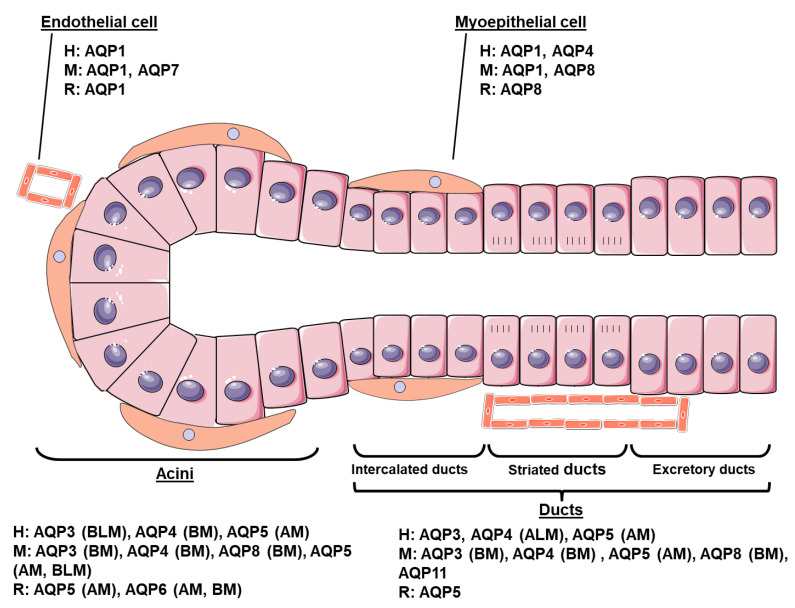
Cellular distribution of aquaporins (AQPs) in salivary glands (SG). Adult SG consist of acinar, ductal and myoepithelial cells. Acinar cells organize into secretory acini structures. Ductal cells organize into intercalated, striated and excretory ducts. Myoepithelial cells surround acini and intercalated ducts. Each cell type expresses some AQPs with particular subcellular localization. H: human; M: mouse; R: rat. AM: apical membrane; ALM: apicolateral membrane; BM: basal membrane; BLM: basolateral membrane.

In rat parotid and submandibular glands, AQP1 is mainly located in erythrocytes and endothelial cells [29,30]. No AQP3 and AQP4 expression has been detected in rat adult SG [18,20]. AQP5 is distributed to acinar apical membrane of the rat submandibular gland [18,22]. In addition, contradictory outcomes of data analysis exist concerning the presence or absence of AQP5 in the ducts [29,31]. However, this localization does fit with the water-impermeable characteristics of ductal cells [32] taken into account in the current model of saliva secretion [1,3]. AQP6 expression was identified in rat parotid acinar cell secretory granules and plasma membranes [16,33]. Furthermore, AQP8 expression is confined to myoepithelial cells [23,31]. Figure 1 recapitulates the cellular distribution of AQPs in adult human, rat and mouse SG.

## 3. Physiological Function of the AQPs in SG

Both sympathetic and parasympathetic nervous systems innervate SG and control SG function through the release of their respective neurotransmitters, adrenaline and acetylcholine, binding to β-adrenergic and M1 and M3 muscarinic receptors, located respectively at the plasma membrane of SG acinar cells [34,35]. In opposition to sympathectomy, parasympathectomy significantly decreased SG AQP5 protein levels without affecting mRNA levels [36], through a post-transcriptional mechanism involving protein degradation in autophagosomes and/or lysosomes [22,37]. The neural signal sent to the submandibular glands via the parasympathetic nerve innervating submandibular glands, i.e., the chorda tympani nerve, was suggested to maintain certain AQP5 expression [22]. Sympathetic activation leads to the release of adrenalin and subsequent activation of intracellular signaling cascade, leading to cyclic AMP (cAMP) increase and a subsequent increase in AQP5 RNA levels and translocation of AQP5 to the cell apical membrane [22,38,39]. Indeed, the injection of isoproterenol, a ß-adrenergic agonist, increased both *Aqp5* mRNA and protein expression, as well as exocytotic translocation of AQP5 from secretory granules to the plasma membrane in mouse parotid glands [22]. Protein kinase A, involved in the cAMP signaling pathway induced by ß-adrenergic stimulation during sympathetic nerve activation, leads to AQP5 phosphorylation, a post-translational modification, on Ser-156 in human and Thr-259 in mouse [22]. AQP5 phosphorylation does not appear to be markedly involved in AQP5 intracellular trafficking [22]. Ser-156 phosphorylation could be involved in constitutive AQP5 membrane expression, while Thr-259 phosphorylation could regulate AQP5 diffusion within the cell membrane [22,40]. M1 and M3 muscarinic receptor (M1R, M3R) activation leads to inositol triphosphate release and intracellular Ca^2+^ increase [41] that can promote AQP5 trafficking to the SG acinar apical membrane. The regulation of SG AQP5 expression under normal and pathological conditions has been reviewed elsewhere [22].

The identification of AQP1 in myoepithelial cells and endothelial cells of the microvasculature suggest a role in salivary fluid production, allowing water to flow from the vascular lumen to the SG [19]. However, this hypothesis was not corroborated in *Aqp1* knockout mice that exhibited unimpaired saliva flow [42]. In addition, despite their expression in SG, neither AQP4 nor AQP8 is involved in the salivation process as both *Aqp4* and *Aqp8* knockout mice did not display decreased pilocarpine-stimulated saliva secretion as compared to wild-type mice [16]. As many knockout animals do not exhibit an obvious phenotype until homeostasis is disturbed and can present compensation mechanisms, further experiments remain to be performed to fully assess the role of these AQPs in salivary secretion. AQP5 is the sole AQP that has been shown to play a key role in saliva production [14,15]. Indeed, *Aqp5*-null mice presented a 60% reduction in pilocarpine-induced saliva, associated with increased saliva viscosity and saliva hypertonicity [15]. Furthermore, the substantial decrease (over 60%) in *Aqp5*-null mouse SG acinar cell membrane water permeability in response to hypertonicity-induced cell shrinkage and hypotonicity-induced cell swelling indicated that AQP5 plays a major role in acinar cells water permeability and saliva secretion [14].

**Figure 2:**
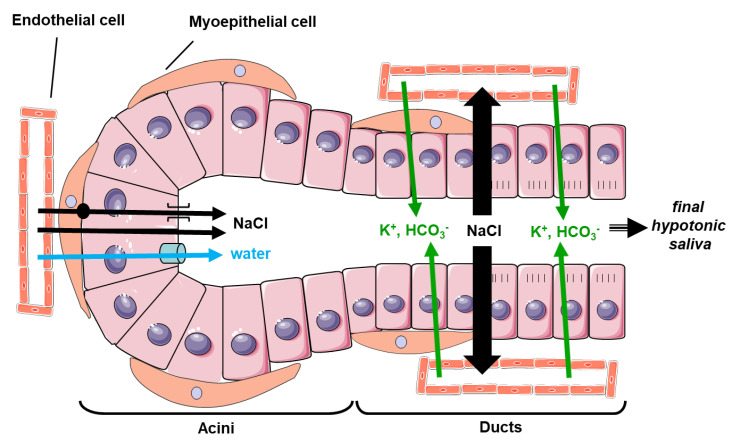
Role of AQP5 in saliva secretion. The saliva secretion process can be subdivided in two phases. In a first phase, SG acinar cells secrete a primary isotonic fluid. This fluid enriched in sodium chloride (NaCl) creates an osmotic gradient. The gradient drives the flow of water to the acini lumen through AQP5 expressed at the apical membrane of acinar cells. AQP5 thereby ensures the trans-cellular permeability and water movement. In a second phase, the ductal cells modify the primary saliva composition by reabsorbing part of NaCl and secreting some bicarbonate (HCO_3_^−^) and K^+^. This mechanism leads to the release of a final hypotonic saliva into the oral cavity.

Considering the relative water impermeability of the ductal cells [32], the physiological role of AQP5 expressed in intercalated ducts from both rat and mice remains to be clarified as it does not fit the current model of saliva secretion [3]. Indeed, in the current two-step model of saliva secretion, SG acinar cells secrete an important concentration of sodium chloride into the acini lumen, leading to the formation of trans-epithelial sodium chloride gradient, subsequent trans-epithelial water transport from the acini to the acini lumen and the formation of a primary isotonic fluid. In the second step of the model, the ductal cells modify the primary isotonic fluid composition by reabsorbing part of the sodium chloride and secreting bicarbonate, leading to a final hypotonic saliva flowing into the mouth cavity [3] (Figure 2).

In addition to the major involvement of AQP5 in saliva secretion, AQP5 and other AQPs might likely be implicated in cell proliferation and migration, neuronal signaling, adhesion and apoptosis occurring during SG embryonic development [18,26,43,44]. The individual role of AQPs in SG embryonic development requires further studies.

## 4. Involvement of AQPs in SG Pathologies

**Figure 3:**
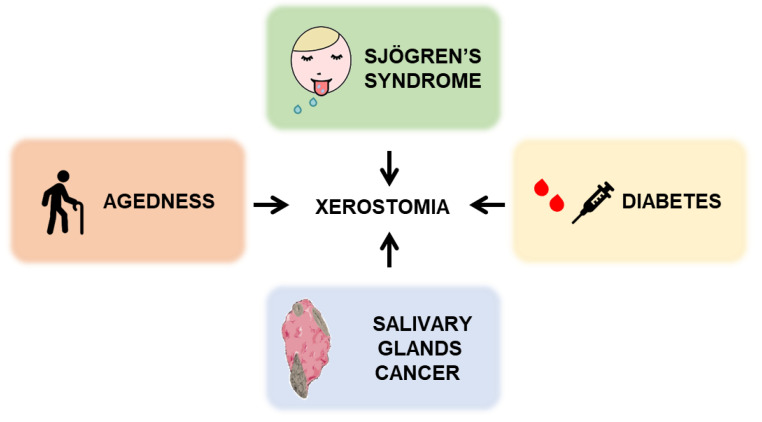
Pathologies leading to xerostomia. Sjögren’s syndrome, diabetes, SG cancer and senescence are often associated with a significant reduction in saliva flow that could partly result from AQP5 expression and/or trafficking.

Since the discovery of AQP water channels, ensuring water permeability across the cellular membrane, studies have contributed to deepen our understanding of their involvement in various physiological conditions, as well as pathological conditions, characterized by disruption in both water homeostasis and transport. In the context of SG physiology, several investigations using AQPs knockout mice have corroborated the specific role of AQP5 as the principal path for transcellular fluid movement in SG acinar cells and its major involvement in saliva secretion [15]. Several pathological conditions affecting SG function have been shown to lead to xerostomia and modified AQP5 expression within SG [31] (Figure 3).

### 4.1. Sjögren’s Syndrome

Sjögren’s syndrome (SS) is a chronic autoimmune disorder characterized by an important lymphocytic infiltration of exocrine glands, resulting in partial destruction of exocrine gland parenchyma leading to their hypofunction [45]. Among the exocrine glands, the salivary and lacrimal glands are the most affected [46]. The pathogenesis of SS is multifactorial and can be subdivided in three phases. In the first phase, the environmental factors, such as viral infection, in association with genetic predisposition and sex hormone imbalance [47], could promote the deregulation of SG epithelial cells (SGECs) and the activation of the innate immune system [48]. In turn, the activation of the innate immune system causes injury of SGECs that activates apoptosis and the presentation of intracellular autoantigens on blebs’ surface [49]. In the second phase, the subsequent proinflammatory response induces the activation of the adaptive immune system, e.g., T and B-lymphocytes. In the third phase, the B-cells proliferate and produce autoantigen-specific antibodies such as anti-SS-A/Ro, anti-SS-B/La [50], anti-muscarinic receptor 3 (anti-M3R) [51] and anti-AQP5 [52]. The formation of the resulting immunocomplexes further exacerbates the immune response and induces a “vicious circle” that culminates in a severe chronic disease. The presence of lymphocytic foci in SG tissue [53] and the formation of central germinal-like structure is a common feature observed in SS biopsy [54]. Although the principal cause of SG hypofunction remains unclear in SS, the characteristics of the disease suggest the involvement of inflammation, acini destruction as well as altered AQP expression and/or localization. A link between inflammation and altered AQP expression, in particular AQP5, has been hypothesized [55,56]. Among the long list of cytokines that have been involved in SS, some have been linked with altered AQP5 expression/localization. IFN-γ, a cytokine produced during all three phases of SS development, induces SG apoptosis, expression of several chemoattractant cytokines and promotes the antigen-presenting activity of SGECs [57,58,59]. *Ifn-γ* gene deficiency prevents the development of the disease in a SS mouse model [60]. Moreover, IFN-γ expression resulting from programmed death ligand-1 (PD-L1) has also been shown to induced anti-M3R antibodies and decreased AQP5 expression in a mouse model of SS [61]. The increased levels of B7 family costimulatory member B7-H3 (CD276) in both serum and SGEC from SS patients were shown to increase the activity of the NF-kB pathway, promote inflammation and decrease AQP5 expression in SGEC [62]. Other studies have highlighted the role of the Tumour Necrosis Factor-α (TNF-α) in SS. Indeed, TNF-α levels are increased in serum and SG from SS patients [63]. In addition, targeted TNF-α overexpression drives mouse SG inflammation [64] and TNF-α treatment of human SG acinar cells induces a significant downregulation of AQP5 expression [65]. Furthermore, the injection of neutralizing antibodies against TNF-α in non-obese diabetic (NOD) mice reduced SG inflammatory foci and increased AQP5 protein expression [66]. Transforming growth factor ß (TGF-ß), interleukin-17 (IL-17) and interleukin-7 (IL-7) also play a role in SS. Indeed, impaired TGF-ß receptor signaling in mice SG resulted in an inflammatory disorder resembling SS, due to SG inflammation and modified AQP5 distribution [67]. *Il-17* overexpression triggers SG inflammation and SG hypofunction in mice [68], while blocking IL-17 results in decreased inflammation and saliva secretion [69]. IL-17 has been recently reported to play a role in epithelial–mesenchymal transition in SGECs from SS patients [70]. Vasoactive intestinal peptide (VIP) administration to NOD mice protects SG against injury and secretory dysfunction by downregulating *Il-17* expression and upregulating *Aqp5* expression [71]. Blocking IL-7-induced levels reduced SG inflammation and hypofunction [72], and upregulated AQP5 expression [73]. Treatment of G-protein-coupled formyl peptide receptor 2 (*ALX/FPR2*) knockout mice with lipopolysaccharides D1 induced SG inflammation and hypofunction, and decreased expression in M3R and AQP5 proteins [74]. While the lipid mediators lipoxin A4 and resolving D1 were able to play a protective role against SG inflammation in this animal model, it remains to be established whether they are capable of restoring M3R and AQP5 expression. All together, these data corroborate the hypothesis of a link between inflammation and altered AQP5 expression/localization [55,56].

Regarding the altered SG AQP expression/localization in SS, some studies have observed a reduced expression of AQP1 [75] and AQP4 [21] in SG from SS patients. On the other hand, a knockout mice model has shown that the inactivation of each of those genes is not associated with a reduction in saliva secretion [76]. However, due to the inherent complexity of knockout animal models and compensation mechanisms encountered in such models, additional experiments will be required to fully understand the role of these AQPs in SG function. In contrast, the *Aqp5*-null mice models display a severe reduction in saliva secretion and SG acinar cell water permeability [14]. AQP5 immunolabeling of SG sections from several animal models for SS and from some SS patients revealed an aberrant expression and/or localization of AQP5 in acinar cells, with a predominant basolateral membrane and/or intracellular localization instead of a typical normal apical membrane localization [56,77,78,79,80,81]. Conversely, SS patients have been described as displaying normal AQP5 localization within SG acinar cells [82,83]. The apparent inconsistencies observed in terms of AQP5 localization in SG from SS patients are likely due to differences in the analytical method used, sensitivity, nature and specificity of the anti-AQP5 antibodies. Additional studies also indicated that aberrant AQP5 localization observed in SG from SS mice models likely results from the presence of inflammatory infiltrates [55,56]. Furthermore, SG hypofunction of SS patients could also partly result from the presence of anti-M3 muscarinic receptor antibodies [84] inhibiting AQP5 trafficking [85], and/or a deficit in inositol triphosphate receptor involved in intracellular calcium release [83]. More recently, the presence of anti-AQP antibodies in blood samples from SS patients has been incriminated in disease manifestations. Indeed, SS patients presenting autoantibodies against the extracellular domain of AQP8 and AQP9 (detected with a higher frequency—39%) or against AQP1 and AQP3 (detected at lower frequency) displays more severe xerophthalmia as compared to control individuals [86]. Moreover, the presence of anti-AQP5 antibodies is considered to be directly linked to SG hypofunction [87,88]. Overall, the discovery of anti-AQP antibodies, and in particular anti-AQP5 antibodies, may offer additional useful biomarkers for SS diagnosis.

The integrity of acini and tight junctions (TJs) between epithelial cells is essential for the formation of apical and basolateral polarity. Indeed, TJs are made of protein complexes, e.g., claudin-4, occludin and zonula occludens (ZO)-1 that define cell polarity and regulate paracellular flow of ions and water [89]. Emerging evidence has shown that TJs protein expression can be modified in response to cytokines under inflammatory conditions [90,91]. In SG from NOD mice, TJs proteins are significantly downregulated at the apicolateral membrane of SGECs, resulting in disruption of TJs barrier [92]. In SS, the altered AQP5 distribution observed at the basolateral membrane or cytoplasm of SG acinar cells may result from a loss TJs protein complexes [93].

In conclusion, in SS, multiple observations have brought to light the role of inflammation in the alteration of AQP5 expression, trafficking and/or localization in SG, as well as the production of autoantibodies against AQPs that consequently could altogether participate to SG hypofunction.

### 4.2. Radiotherapy for Head and Neck Cancer

Head and neck cancer include a heterogeneous group of tumours that affect the upper aerodigestive tract, paranasal sinuses and salivary and thyroid glands. For most patients, surgery and radiotherapy are the major treatment modalities [94]. Radiotherapy often results in a loss or reduction of the sense of taste, acute mucositis of both oral cavity and pharynx, pain and decreased saliva secretion [95]. The loss of AQP5 expression could participate in the development of severe xerostomia in patients undergoing radiotherapy. Rat submandibular glands subjected to a low-dose radiation displayed decreased AQP5 expression [96,97,98] and impaired AQP5 trafficking [99], as compared to the sham-irradiated animals. Radiation treatment of organotypic cultures from mice SG showed a time-dependent decrease in AQP5 expression [100]. Decrease in AQP1 expression within the endothelium as well as AQP5 in acinar and ductal cells has been observed along with decreased saliva secretion [29,98,101]. Long-term administration of pilocarpine to irradiated mice has a beneficial effect in terms of saliva secretion [102]. Alpha-lipoic acid also rescued radiation-induced SG hypofunction in rats [97].

### 4.3. SG Cancer

**Figure 4:**
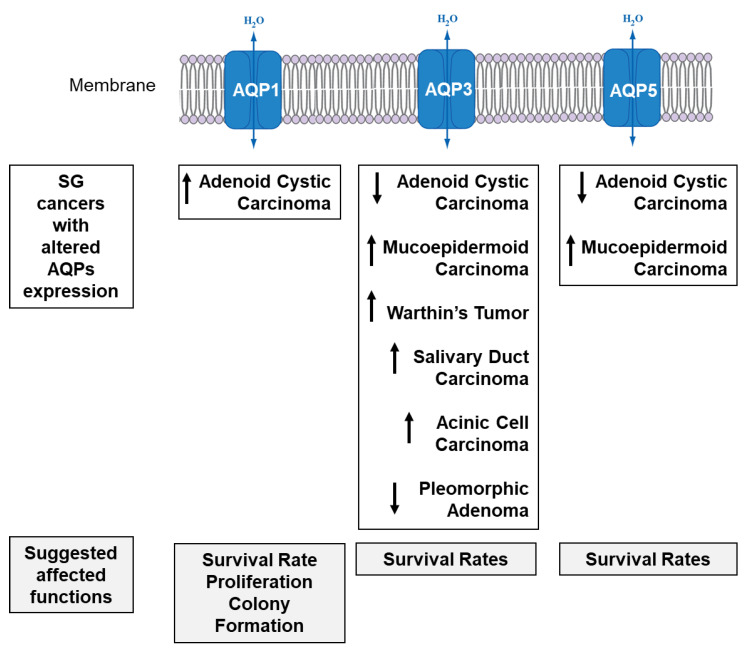
SG cancers with altered AQP1, AQP3 and AQP5 expression and their potential effects on functions. Top of diagram: List of different SG cancer types reported with an increase (↑) or decrease (↓) in the expression of AQPs when compared to their normal tissues. Bottom of diagram: A list of possible effects on cancer function due to altered AQPs expression.

Each AQP type may participate in a specific carcinogenesis process (Figure 4), including migration, invasion, metastasis, proliferation and drug resistance, which affect the prognosis of specific cancer type(s) [103]. AQPs play an essential role in at least three of the original hallmarks of cancer (angiogenic, invasion and metastasis) and in one of the cancer enabling-characteristics, as it contributes to and facilitates the transport of reactive oxygen species, which in turn increase tumor-promoting inflammation and induce genome instability and mutations [104]. Indeed, AQP1 induces angiogenesis [105,106,107], AQP3 stimulates cellular migration, proliferation or invasion [104,105], AQP5 expression correlates to cancer proliferation and migration [106,107,108,109,110], and AQP5 and AQP9 regulate chemotherapy resistance [111].

Although there are several studies related to aquaporins in normal SG tissues, research on SG cancers still remains limited. In SG adenoid cystic carcinoma (ACC), AQP1 was found to be hypomethylated with increased protein expression when compared to non-cancerous tissues [112]. In a study showing significant hypomethylation but only a trend toward increased *Aqp1* mRNA expression, there was an association between AQP1 hypermethylation and the improved overall survival rate, but no relation was found with recurrence- or metastasis-free survival between *Aqp1* mRNA level and prognosis [113]. Additional studies will be required to increase the number of patients and draw clear conclusions. Ha and colleagues reported an increase in cellular proliferation and colony formation with AQP1 transfection in vitro [112], with no significant role in cell migratory or invasive capability [113]. ACC had low or no AQP3 and AQP5 expression when compared to normal tissues, which might be due to a downregulation of these proteins during de-differentiation and cancer development [114,115]. In SG mucoepidermoid carcinomas, AQP1 was only present in the vascular endothelium, while AQP3 was found in epidermoid and mucous cells and AQP5 in mucous cells [19,115]. AQP3 expression was reported to be higher in Warthin’s tumor, salivary duct carcinomas and in acinic cell carcinomas, while low in pleomorphic adenomas [115].

### 4.4. Agedness and Diabetes

Agedness induces a gradual decline in saliva production in humans, mice and rats [116,117,118]. In addition, agedness was reported to induce a decrease in AQP5 levels [117,118,119] and in acetylcholine-induced increase in AQP5 levels, independent of a modification in M3R number [120].

Diabetes, a disease estimated to reach a prevalence of 4.4% and affecting about 366 million people worldwide in 2030 [121], induces xerostomia [122,123]. Although type 1 diabetes in rat and mouse animal models displays reduced saliva production, divergent data exist concerning a modification in AQP5 expression, localization and muscarinic agonist-induced translocation [79,124,125]. It is noteworthy that maternal type 1 diabetes induced postnatal changes in the development of rat SG in offspring, including decreased AQP5 expression [126]. Rats with type 2 diabetes displayed decreased saliva flow, inflammatory cell infiltration of SG and decreased AQP5 expression [127]. Further studies are required to fully assess the role of AQP5 in diabetic xerostomia.

## 5. Therapeutic Strategies Aiming at AQPs to Treat Xerostomia

**Figure 5:**
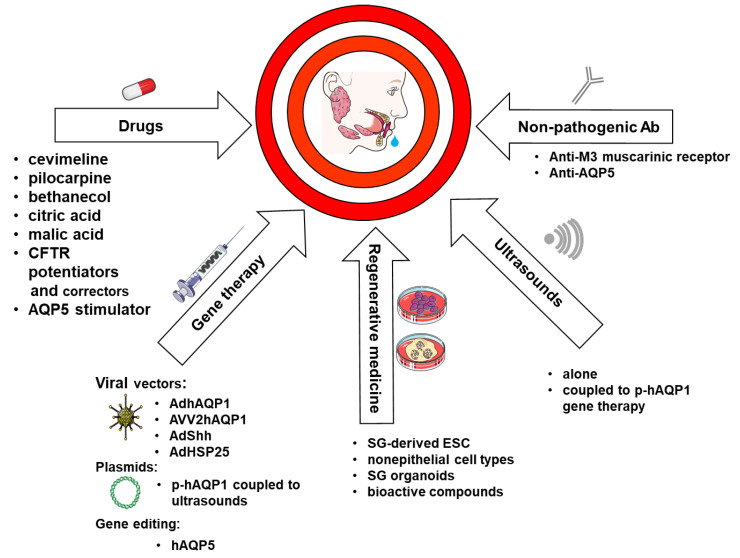
Current and future therapeutic strategies, including AQPs, to treat xerostomia. The diagram recapitulates different treatment strategies (drugs, gene therapy, regenerative medicine, ultrasounds, non-pathogenic Ab) to restore loss of function, to prevent or to treat the common symptoms and/or consequential xerostomia.

The etiology of xerostomia, or dry mouth, arises from iatrogenic (drugs, head and neck radiotherapy, chemotherapy), developmental (SG agenesis or atresis), pathological (sialolithiasis, sialadenitis), immunological (autoimmune SS), infectious (viral infections), metabolic causes (diabetes) or others causes (cystic fibrosis, aging, amyloidosis, hemochromatosis, Wegener’s disease) [128]. Due to its involvement in the molecular mechanisms of saliva secretion, AQP5 became an additional therapeutic target for the treatment of xerostomia. Figure 5 summarizes current and future treatment aiming at AQPs to treat xerostomia.

### 5.1. Drugs

Xerostomia may be improved by drugs promoting the activation of any protein participating in the intracellular signaling cascade, leading to saliva secretion upon cholinergic and ß-adrenergic nerve stimulation. M3 and M1 muscarinic agonists, such as cevimeline and pilocarpine, are commonly used to treat xerostomia of various origins in human [129,130,131,132]. Bethanechol, a M3 muscarinic agonist resistant to cholinesterase, prevented a decrease in stimulated saliva flow in patients undergoing head and neck radiotherapy or chemoradiation [133,134]. In contrast, with cevimeline and pilocarpine, the beneficial effects of bethanechol remain to be assessed in larger randomized clinical trials [132]. Muscarinic activation was reported to promote calcium-induced AQP5 trafficking to acinar cell apical membranes in rat SG [135,136]. Furthermore, in mice SG subjected to radiation, cevimeline prevented both xerostomia and a decrease in AQP5 expression [137] and re-established AQP5 localization to the acinar cell apical membrane [24,138].

Citric and malic acids, acting through taste buds and parasympathetic pathways, conferred some beneficial effects for xerostomia but increased the risk of dental erosion and caries [128]. However, the effects of both citric and malic acids on AQP5 localization and expression remain to be documented under xerostomic conditions.

Modulators of cystic fibrosis transmembrane conductance regulator (CFTR) have been shown to restore saliva secretion. Indeed, CFTR potentiator VX770 and CFTR corrector C18 administration restored saliva secretion in NOD mice, a widely used animal model for SS, and C18 had a similar effect in mice overexpressing BMP6, another animal model for SS [139]. In addition, both molecules decreased SG inflammation and restored AQP5 protein expression as well as transepithelial water triggered by muscarinic stimulation in SG from NOD mice [139]. Eluforsen, a RNA oligonucleotide, restored saliva secretion in female F508del-CFTR mice, an animal model of cystic fibrosis [140]. Clinical trials have shown that eluforsen improved CFTR activity [141] and questionnaire-revised respiratory symptom score [142] in F508del-CFTR homozygous patients. However, further clinical trials will have to assess the beneficial effect of eluforsen on SG function of F508del-CFTR homozygous patients. Further studies will be required to assess the effects of these CFTR-targeted drugs on restoration of salivation and on AQP5 protein expression and distribution in SG in patients suffering from other physiopathological conditions leading to xerostomia (e.g., SS patients).

Other compounds have also been found to increase saliva and AQP5 expression, such as asthaxanthin [119] and Ixeris dentata extract [117,124]. However, the beneficial effects of these other compounds will have to be properly assessed. In addition, future development of drugs enhancing AQP5 activity may offer extra therapeutic leverage to alleviate xerostomia.

### 5.2. Ultrasounds

SG from an MRL/MpJ/lpr/lpr (MRL/lpr) SS mouse model, subjected to low-intensity pulsed ultrasounds, displayed restored saliva secretion and AQP5 expression, as well as reduced inflammation [143]. Further studies will have to be carried out to assess whether low-intensity pulsed ultrasounds could be used as an efficient non-invasive therapy for the treatment of xerostomia in patients having undergone radiotherapy or suffering from SS.

### 5.3. Non-Pathogenic Antibodies

Engineered human monoclonal IgG antibodies, with effector function neutralized, could be used to bind to M3R or AQP5 and prevent complement and cellular cytotoxicity of autoantibodies directed against these proteins. The development of such new therapeutic agents could be useful to treat xerostomia in SS patients. The proof-of-concept of such new therapeutic approach is currently being evaluated using non-pathogenic anti-AQP4 antibodies (aquaporumab) for the treatment of neuromyelitis optica characterized by the presence of autoantibodies against AQP4 [144,145].

### 5.4. Gene Therapy

Due to their quite unique structural characteristics, i.e., encapsulation and accessibility via ductal opening in the mouth cavity, SG represent interesting targets for gene therapy [146]. Adenovirus-mediated *hAqp1* gene delivery restored saliva secretion in irradiated rat [147], mouse [148] and minipig SG [149]. Adenovirus-mediated *hAqp1* gene delivery to non-human primate-irradiated SG was well tolerated but inconsistently improved the SF function [150]. Adenovirus-mediated *hAqp1* gene transfer restored both salivary and lacrimal secretion and decreased local and systemic inflammation in a SS mouse model induced by BMP6 overexpression [151]. A phase I clinical trial (NCT00372320) revealed that adenoviral vector encoding hAqp1 improved saliva secretion in six out of eleven patients suffering from SG hypofunction post-irradiation therapy [152], and that this improvement persisted for 3 to 4.7 years post-administration [153]. Five of the six responders also showed improvements in subjective perception of oral dryness and amount of saliva present in the mouth by visual scale assessment [152]. The patients responding to the treatment were shown to display modest immune reactivity following gene transfer, as compared to the patients not responding to the treatment [153]. This proof-of-concept study remains to be corroborated by a placebo-controlled clinical trial using a large number of subjects.

Adeno-associated virus (AAV2)-mediated *hAqp1* gene delivery to irradiated minipig SG also restored saliva secretion [154] and induced long-term gene expression in mice SG without inducing significant adverse effects [155]. As AAV2-based vectors exhibit lower immunogenicity and more stable expression than adenoviral vectors, they are more promising gene therapy alternatives for xerostomia. In this line of thought, it is interesting to acknowledge an ongoing phase I clinical trial that is currently assessing the safety of a single administration of AAV2*hAqp1* to one parotid SG in patients with irradiation-induced parotid salivary hypofunction (NCT02446249). The future will tell us if AAV2-based vectors encoding *hAqp1* gene therapy continue to hold their promise as a potential strategy for treatment to tackle radiotherapy-induced xerostomia. Furthermore, genetic modifications of adeno-associated viral vectors may facilitate the success of adeno-associated virus-based gene therapy [156].

Viral gene delivery of sonic hedgehog has been shown to restore salivary secretion and AQP5 expression in irradiated mouse and minipig SG [157,158,159]. In addition, viral delivery of heat shock protein 25 showed similar effects in irradiated mouse SG [160].

Low-intensity pulsed ultrasounds’ delivery of plasmid DNA encoding hAqp1 to SG from irradiated minipig also improved saliva secretion [161].

Clustered Regularly Interspaced Short Palindromic Repeats (CRISPR)-CRISPR-associated protein 9(Cas9) (CRISPR-Cas9) homologous-directed repair system allowing the integration of the cytomegalovirus promoter upstream of the endogenous *Aqp1* gene in HEK293 and MDCK cells resulted in higher AQP1 protein expression and transepithelial water permeability [162]. Gene editing technologies, including CRISPR-Cas9, have been used in clinical trials for either, and most frequently, ex vivo modification of cells and reinfusion into the patient, or, less frequently, for direct in vivo cell modification by injection of the gene editing system [163,164]. Efforts have been devoted to reduce off-target effects, one of the major concerns of gene editing technology [163]. As major SG can be reached by retrograde ductal infusion through their main duct openings from the oral cavity, SG represent additional targets for in vivo gene editing. In the future, gene editing may be used to treat xerostomia or other SG pathological conditions, for example by increasing AQP5 expression in SG acinar cells or even by simultaneous multigenic gene editing.

Overall, additional placebo-controlled trials using a large intervention group are necessary to assess the benefits of gene delivery (such as, for example, *hAqp1*) in patients suffering from xerostomia resulting from radiotherapy or SS. The benefit of gene therapy mostly relies on the development of safer and more efficient viral vectors, non-viral methods ensuring gene delivery and gene editing technologies.

### 5.5. AQPs and Regenerative Medicine

Several AQPs have been shown to play a role in organ regeneration, including AQP1 in kidney regeneration [165] and in neuronal regeneration [166], and AQP8, AQP9 and AQP11 in liver regeneration [167].

A well-studied model for SG regeneration is the experimental ligation of the main excretory ducts, which leads to the apoptosis of acinar cells, shrinkage of the remaining acinar cells and proliferation of ductal cells [168,169]. Reopening of the ligated salivary main excretory duct stimulates the repopulation and restoration of the gland back to its normal morphology. As AQPs are essential molecules during SG development, they have been used as markers for SG regeneration [22,170,171]. In mouse experiments, the expression level of AQP5 decreased during the ligation period, as measured by western blotting. The remaining AQP5 expression was still localized at the apical membranes of the remaining shrunken acinar cells [172]. After reopening of the ligated duct, there was a gradual restoration of the expression of AQP5 with localization to the apical membrane of the acinar cells and in intercalated ductal cells [173]. Another study reported a transient reduction in AQP5 expression after ligation removal followed by restoration of the normal expression and localization at the apical, basal and lateral membranes of the SG cells [171]. Results have been variable between different animal and SG models, or to methods of duct ligation [174].

Several studies have utilized AQPs expression, especially AQP5, as a marker to assess SG regeneration following an experimental therapeutic intervention. The increase in AQP5 expression was used as a marker for the reversal of xerostomia caused by the autoimmune sialadenitis associated with Sjögren’s syndrome, using a low-intensity pulsed ultrasound [143]. AQP5 has been used as a salivary acinar cell marker and displayed elevated gene expression in SGs of NOD mice treated with either spleen or mesenchymal stem cells [175,176]. Elevated gene expression of AQP1, AQP4 and AQP5 have been measured in mice models of xerostomia treated with a cell extract from either bone marrow or mesenchymal stem cells, which reduced salivary focus score, and restored saliva and tear flow rates [177,178,179].

Elevated AQP5 expression was noted after rescuing damaged SGs using adipose-derived mesenchymal stem cells following radioiodine therapy, which is usually used for patients with thyroid cancer [179]. Using an irradiation-injured SG mouse model, elevated AQP5 expression levels were consistently observed following treatment with a bone marrow cell extract in a mouse model in which SGs were injured by either a single- or fractionated-dose of irradiation [180,181,182]. Recently, the use of cell extract from human labial minor SGs was successful in preserving AQP5-positive acinar cells and restoring salivary function to irradiated-injured SGs of mice [183].

In the field of tissue engineering, AQP5 has been used as a marker for functional analysis of bioengineered SGs. Ogawa and colleagues reported the orthotopic transplantation of a bioengineered SG germ as a regenerative organ replacement therapy [184]. Their bioengineered SG developed into a mature gland with acinar cells expressing AQP5 at the apical membrane. In a subsequent study, the same group of researchers used embryonic stem cells to develop functional SGs that expressed AQP5 at the apical membrane of acinar-like cells both in-vitro and in-vivo [185]. Our group has investigated for years the methods of isolating and culturing human salivary epithelial cells, and particularly acinar cells for tissue engineering purposes [186]. Our work has progressed steadily, but major hurdles remain to be solved. When the extracellular matrix is preserved, as in our proposed “salivary slice culture model”, human acinar cells retained their AQP5 expression and apical location [187]. However, when these human salivary cells were enzymatically digested and plated on three-dimensional (3D) gels or in polarized cell monolayers, AQP5 expression was either lost or its localization was shifted to the cytoplasm [188,189,190]. Recently, we have devised a new strategy by using a serum-free scalable suspension culture system that grows partially digested human salivary tissue filtrates composed of acinar and ductal cells attached to their native extracellular matrix components, while retaining their 3D in vivo spatial organization [191]. We demonstrated that aggregates of cells remained proliferative and continued to express acinar (such as AQP5) and ductal cell-specific markers for 5 to 10 days [191]. We have also reported that two cell surface markers, CD44 and CD166, used in the isolation of human mesenchymal stem cells could be co-localized with AQP5-positive serous (CD44) and mucous (CD166) acinar cells [192]. These cell markers, in combination with AQP5, could potentially be useful in tracking cell transplants to regenerate SGs in patients, although our current data reported a low rate of engraftment (1%) of marrow-derived stem cells into SGs [193].

Another method for using AQPs in regenerative medicine was introduced by Kato et al., in which multiple cycles of freezing and thawing allowed to select/concentrate a particular cell type or cell lineage that expressed one or several AQPs from cells or cell lineages devoid of AQP expression [194]. This method relies on a differential resistance to membrane damage caused by ultra-quick freezing (higher in cells expressing AQPs than in cells that did not express AQPs).

## 6. Conclusions

Saliva is constantly produced and secreted into the oral cavity by SG. AQPs, and in particular AQP5, plays an essential role in the saliva secretion process. Numerous studies have reported the involvement of AQPs in pathological conditions affecting SG. Therefore, a better understanding of the molecular mechanisms of action and properties of AQPs is the key to appreciate their contribution to SG homeostasis and pathological conditions affecting SG, as well as to developing new therapeutic approaches. The scientific evidence acquired so far has indicated that AQPs may represent valuable targets for multiple therapeutic applications to treat SG xerostomia. As such, drugs, *Aqp* gene therapy, as well as tissue engineering and stem-cell based therapy, represent promising therapies to treat xerostomia arising from various origins. Further studies are still required to move forward to the development of curative strategies for the treatment of xerostomia.
